# Early Serum S100B and Neurophysiological and Structural Brain Abnormalities in a Prospective Cohort of Preterm Infants

**DOI:** 10.3390/children13101403

**Published:** 2026-10-14

**Authors:** Nishankul Bozhbanbayeva, Abay Kussainov, Indira Suleimenova, Olga An, Gulmira Altynbayeva, Saltanat Sairankyzy, Sandugash Zhexengul, Indira Adilbekova

**Affiliations:** 1 Department of Neonatology, Asfendiyarov Kazakh National Medical University, Almaty 050012, Kazakhstan; bozhbanbaeva.n@kaznmu.kz (N.B.); kusainov.abai67@mail.ru (A.K.); suleymenova.i@kaznmu.kz (I.S.); sairankyzy.s@kaznmu.kz (S.S.); sandu-1991@mail.ru (S.Z.); adilbekova.i@kaznmu.kz (I.A.); 2 JSC Scientific Center of Pediatrics and Pediatric Surgery, Almaty 050023, Kazakhstan; gulmira_altynbaeva@mail.ru; 3 Department of Pediatrics with a Course in Neonatology, Kazakhstan Medical University “KSPH”, Almaty 050060, Kazakhstan

**Keywords:** preterm infants, S100B, electroencephalography, cranial ultrasonography, intraventricular hemorrhage, periventricular leukomalacia

## Abstract

**Highlights:**

**What are the main findings?**

• Higher early neonatal serum S100B concentrations were associated with greater EEG abnormality severity in preterm infants, whereas no significant associations were observed with IVH or PVL severity.
• The association between S100B and EEG abnormality severity remained evident in a sensitivity analysis restricted to infants with a postmenstrual age of ≤36 weeks using a preterm-specific EEG grading system.

**What are the implications of the main findings?**

• Early serum S100B may provide complementary information on neurophysiological disturbance when interpreted alongside EEG as part of multimodal neurological assessment in preterm infants.
• The absence of an association with early ultrasound-defined IVH or PVL suggests that S100B and cranial ultrasonography may capture different aspects of early neurological involvement, warranting longitudinal evaluation with later neuroimaging and neurodevelopmental follow-up.

**Abstract:**

**Background:** Early identification of brain dysfunction in preterm infants remains challenging because cranial ultrasonography (CUS) may not capture early neurophysiological abnormalities. Serum S100B has been proposed as a marker of neonatal brain injury, but its relationship with electroencephalographic (EEG) abnormalities in preterm infants remains unclear. **Objectives**: Our objectives were to evaluate the association between early neonatal serum S100B concentrations and EEG abnormality severity in preterm infants and to determine whether similar associations are observed with structural brain injury detected by CUS. **Methods**: This prospective observational cohort included 116 preterm infants from 281 prospectively enrolled neonates. Serum S100B was measured within the first 24 h of life. Conventional multichannel EEG was performed at 10–14 days of life, and CUS was performed serially to assess intraventricular hemorrhage (IVH) and periventricular leukomalacia (PVL). Associations with EEG severity were evaluated using correlation analysis and ordinal logistic regression. A sensitivity analysis was restricted to infants with a postmenstrual age (PMA) ≤ 36 weeks at EEG and used a preterm-specific EEG grading system. **Results**: Higher log10(S100B) concentrations were associated with greater EEG abnormality severity (Spearman’s ρ = 0.49, *p* < 0.001) and remained independently associated after adjustment for gestational age and Respiratory Distress Syndrome (RDS) (adjusted OR 19.95, 95% CI 4.97–80.16; *p* < 0.001). No significant associations were observed between S100B concentrations and IVH or PVL severity. In the PMA-restricted sensitivity analysis (n = 89), the association between S100B and EEG abnormality severity remained significant using the preterm-specific grading system (ρ = 0.54, *p* < 0.001). **Conclusions**: During the early neonatal period, higher serum S100B concentrations were associated with greater neurophysiological abnormality severity in preterm infants, whereas associations with ultrasound-defined IVH or PVL were not demonstrated. The persistence of the S100B–EEG association in the PMA-specific sensitivity analysis supports the robustness of this finding across EEG grading approaches.

## 1. Introduction

Preterm birth is recognized as one of the leading causes of neonatal morbidity and long-term neurodevelopmental vulnerability worldwide. According to recent World Health Organization estimates, approximately 13.4 million preterm births occurred globally in 2020 [1], while demographic projections suggest that prematurity will continue to represent a substantial public health burden in Kazakhstan through 2050 [2,3]. Despite ongoing advances in neonatal intensive care, preterm infants remain particularly vulnerable to systemic inflammation, infectious complications, respiratory instability, and disturbances of early neurodevelopment during a critical period of postnatal adaptation [4].

Early neurological assessment in preterm infants requires complementary approaches to characterize both structural brain injury and functional cerebral abnormalities. Cranial ultrasonography (CUS) remains the principal bedside neuroimaging modality because it enables serial detection of major structural lesions, including germinal matrix–intraventricular hemorrhage (IVH), periventricular hemorrhagic infarction, and white matter injury [5]. While CUS provides information on structural brain abnormalities, electroencephalography (EEG) enables functional assessment of cerebral activity through evaluation of background continuity, cyclicity, symmetry, synchrony, maturational features, and abnormal EEG patterns [6]. Despite the complementary roles of these methods, there remains a clinical need to explore markers that may provide additional information on evolving cerebral disturbances and help identify preterm infants who warrant closer neurological surveillance and longitudinal assessment.

In recent years, increasing attention has been directed toward circulating biomarkers as potential early indicators of neurological injury. Such biomarkers may provide complementary information on evolving cerebral disturbances alongside clinical, neurophysiological, and structural assessments. S100B, a calcium-binding protein expressed predominantly by astroglial cells, has been investigated as a potential biomarker of neonatal brain injury [7]. Evaluating early S100B concentrations alongside functional EEG findings and structural changes detected by CUS may therefore provide a more comprehensive assessment of neurological involvement during the neonatal period, particularly in high-risk populations such as preterm infants [8,9]. However, the relationship between early circulating S100B and neurophysiological abnormalities assessed by conventional EEG, in comparison with structural abnormalities detected by CUS, remains insufficiently characterized in preterm infants. In this context, we aimed to investigate the relationship between early neonatal serum S100B concentrations and neurophysiological and structural brain abnormalities in preterm infants.

## 2. Materials and Methods

Study design and population: This prospective observational cohort study was conducted between November 2024 and February 2026 in the neonatal intensive care units (NICUs) of two tertiary-level perinatal centers in Almaty, Kazakhstan, and was reported in accordance with the STROBE guidelines for observational studies [10]. A total of 281 preterm infants admitted during the study period were prospectively enrolled.

Preterm infants born between 22 and 36 completed weeks of gestation and admitted to the participating NICUs were eligible for inclusion. Written informed consent was obtained from the parents or legal guardians before study participation. Infants with major congenital malformations, chromosomal abnormalities, or confirmed congenital metabolic disorders were excluded.

During hospitalization, participants underwent standardized clinical, laboratory, and neurological assessments. Serum S100B was measured within the first 24 h of life, CUS was initiated on the first day of life and repeated serially during the neonatal period, and EEG was performed at 10–14 days of life. Because EEG assessment required separate written parental informed consent, 116 infants underwent EEG and constituted the analytical cohort for the neurophysiological analyses. Baseline characteristics of infants who underwent EEG and those who did not were compared to assess potential selection bias.

The primary exposure was early neonatal serum S100B concentration. The principal outcome was EEG abnormality severity, while IVH and PVL assessed by serial CUS were evaluated as structural neurological outcomes. All analyses involving the principal study variables were performed in the complete EEG cohort (n = 116), with no missing data for the variables included in the main analyses.

S100B measurement: Serum S100B concentrations were measured in blood samples collected within the first 24 h of life. Measurements were performed independently at a specialized laboratory using the CanAg S100 EIA kit (Fujirebio Diagnostics AB, Gothenburg, Sweden) in accordance with the manufacturer’s instructions. The analytical detection limit was <10 ng/L, with a measuring range of 10–3500 ng/L. S100B was used as the primary neurobiological biomarker in the present study.

Neurophysiological and neuroimaging assessment: Serial CUS was initiated on the first day of life and subsequently performed at 3-day intervals until 10–14 days of life. Examinations were performed through the anterior fontanelle using a microconvex transducer, with the operating frequency adjusted according to infant size and imaging depth. Standard coronal and sagittal planes were obtained to assess the ventricular system, germinal matrix, and periventricular white matter [11,12]. IVH was graded according to the Papile classification [13,14], and PVL according to the de Vries classification [11,15]. Grade I PVL was defined as abnormal periventricular echogenicity persisting for ≥7 days on serial examinations [15]. For statistical analyses, the highest confirmed grade of IVH and PVL observed across serial CUS examinations was used. CUS examinations were performed and interpreted by experienced specialists at the participating clinical centers and subsequently reviewed by a designated responsible specialist.

Conventional EEG was performed at 10–14 days of life using standardized 3-h multichannel recordings acquired with a NicoletOne EEG system (Natus Medical Incorporated, Middleton, WI, USA). A reduced neonatal montage based on the international 10–20 electrode placement system was used, comprising nine scalp electrodes (Fp1, Fp2, C3, C4, Cz, T3/T7, T4/T8, O1, and O2). EEG background abnormalities were graded according to the system described by Murray et al. [16] Given the maturational dependence of EEG characteristics in preterm infants, a sensitivity analysis was performed in infants with a postmenstrual age (PMA) of ≤36 weeks at the time of EEG recording (n = 89). EEG recordings in this subgroup were reassessed using the standardized grading system for conventional EEG in preterm infants described by Pavlidis et al. [17], which incorporates PMA-dependent characteristics of EEG background organization and maturation. Recordings were interpreted at an independent specialized neurophysiology clinic by experienced neurophysiology specialists and subsequently reviewed by a designated senior neurophysiologist before the final report was issued. Assessors interpreting both CUS and EEG recordings were blinded to serum S100B concentrations.

Statistical analysis: For statistical analyses, serum S100B concentrations were log10-transformed to reduce right-skewness and improve their suitability for regression analyses. EEG severity was grouped into three categories in both the Murray and Pavlidis PMA-specific grading approaches: normal/mild (grades 0–1), moderate (grade 2), and severe (grades 3–4). IVH and PVL severity were each categorized as no injury (grade 0), mild injury (grades I–II), and severe injury (grades III–IV). Categories were combined to ensure adequate numbers of infants within each severity group and to improve statistical stability given the small numbers in the extreme severity categories.

Continuous variables were assessed for normality using the Shapiro–Wilk test. As most continuous variables were not normally distributed, data are presented as median and interquartile range (IQR), whereas categorical variables are presented as frequencies and percentages.

Associations between continuous or ordinal variables were assessed using Spearman’s rank correlation coefficient. Comparisons across EEG, IVH, and PVL severity categories were performed using the Kruskal–Wallis test, followed by Dunn’s post-hoc test with Holm correction for multiple comparisons where appropriate. These analyses were also applied in the PMA-specific EEG sensitivity analysis.

EEG abnormality severity was evaluated using ordinal logistic regression. Univariable ordinal logistic regression analyses were initially performed to assess associations between EEG severity and serum S100B concentrations and other clinical and perinatal factors. A parsimonious multivariable model was subsequently constructed to evaluate the independent association between S100B and EEG abnormality severity while accounting for clinically relevant potential confounding. Gestational age was selected as the principal measure of prematurity, and birth weight and birth length were therefore not included simultaneously. RDS was included as a clinically relevant marker of early respiratory morbidity, while duration of Invasive mechanical ventilation (IMV) was not entered simultaneously to avoid redundancy between related respiratory severity variables. The final adjusted model for EEG severity included log10-transformed S100B, gestational age, and RDS.

For the structural outcomes, IVH and PVL severity were evaluated using separate ordinal logistic regression models. Given the small numbers of infants in the severe IVH and PVL categories, the multivariable models were restricted to log10-transformed S100B and gestational age to reduce the risk of overfitting. The proportional odds assumption was evaluated using the Test of Parallel Lines.

Statistical analyses were performed using IBM SPSS Statistics for Windows, version 28.0 (IBM Corp., Armonk, NY, USA). All tests were two-sided, and a *p* value < 0.05 was considered statistically significant.

## 3. Results

### 3.1. Study Population

Among the 281 preterm infants prospectively enrolled in the study, 116 underwent EEG assessment at 10–14 days of life and comprised the final analytical cohort. To assess potential selection bias, baseline demographic and clinical characteristics were compared between infants who underwent EEG and those who did not (Table 1). No statistically significant differences were observed between the two groups in gestational age, birth weight, birth length, or Apgar scores at 1 and 5 min (all *p* > 0.05).

After confirming the comparability of the EEG and non-EEG groups, the baseline characteristics of the final analytical cohort (n = 116) are summarized in Table 2.

The analytical cohort included 116 preterm infants. Respiratory morbidity was common, with RDS diagnosed in 80 infants (69.0%); 59 (50.9%) received CPAP and 37 (31.9%) required IMV. Mild IVH (grades I–II) was identified in 64 infants (55.2%), whereas severe IVH (grades III–IV) occurred in 6 (5.2%). PVL was identified in 26 infants (22.4%), including 21 (18.1%) with grades I–II and 5 (4.3%) with grades III–IV. According to the Murray classification, EEG background abnormalities were categorized as normal/mild in 15 infants (12.9%), moderate in 58 (50.0%), and severe/inactive–ictal in 43 (37.1%).

### 3.2. Association Between S100B and EEG Abnormality Severity

**Figure 1:**
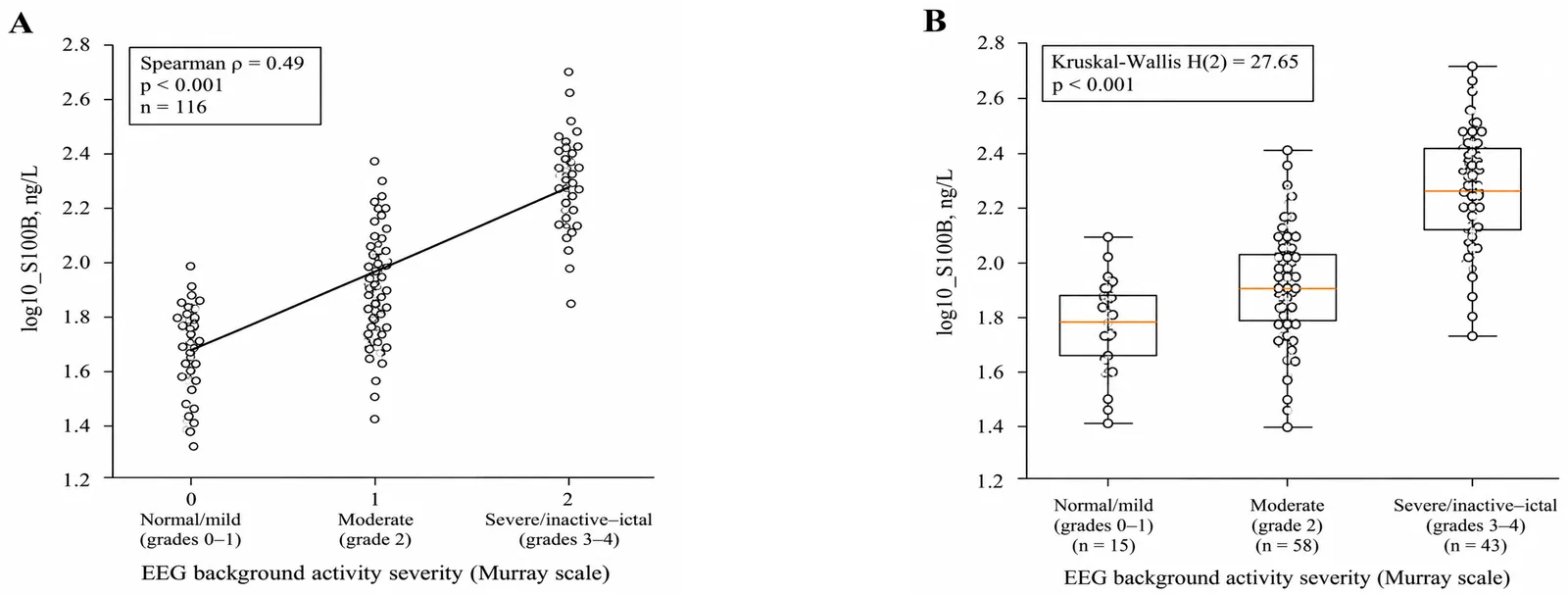
Association between serum S100B concentrations and EEG background abnormality severity according to the Murray classification: (**A**) correlation analysis; (**B**) distribution across EEG severity categories.

Serum S100B concentrations were non-normally distributed, with a median of 85.0 ng/L (IQR 56.2–134.2; range 20.0–642.7 ng/L). Higher serum S100B concentrations were significantly associated with increasing severity of EEG background abnormalities according to the Murray classification (Spearman’s ρ = 0.49, *p* < 0.001; Figure 1A). Log10-transformed S100B concentrations differed significantly across the three EEG severity categories (Kruskal–Wallis H(2) = 27.65, *p* < 0.001; Figure 1B). Median log10(S100B) concentrations were 1.782 (IQR 1.660–1.853) in the normal/mild group (n = 15), 1.841 (IQR 1.701–1.969) in the moderate group (n = 58), and 2.127 (IQR 1.985–2.426) in the severe/inactive–ictal group (n = 43). Dunn’s post-hoc test with Holm correction showed significantly higher S100B concentrations in the severe/inactive–ictal group than in both the normal/mild and moderate groups (both adjusted *p* < 0.001), whereas the difference between the normal/mild and moderate groups was not statistically significant (adjusted *p* = 0.138).

In univariable ordinal logistic regression, higher log10-transformed serum S100B concentrations were significantly associated with greater EEG abnormality severity (OR 26.04, 95% CI 6.75–100.49; *p* < 0.001). The proportional odds assumption was satisfied for the S100B model (Test of Parallel Lines: χ^2^(1) = 0.038, *p* = 0.846). Univariable associations between EEG abnormality severity and other clinical and perinatal factors are presented in Table 3.

In addition to S100B, lower gestational age, lower birth weight and birth length, lower Apgar scores, longer duration of IMV, and RDS were significantly associated with greater EEG abnormality severity in univariable analyses (Table 3). Other evaluated clinical factors were not significantly associated with EEG severity. Estimates for neonatal sepsis and NEC were not obtainable because of the small number of events.

A parsimonious multivariable model was constructed to account for clinically relevant potential confounding while avoiding the simultaneous inclusion of closely related variables. Gestational age was selected as the principal measure of prematurity, and birth weight and birth length were therefore not included in the same model. RDS was included as a clinically relevant marker of early respiratory morbidity, while duration of IMV was not entered simultaneously to avoid redundancy between related respiratory severity variables. The final multivariable model included log10-transformed S100B, gestational age, and RDS (Table 4).

In multivariable ordinal logistic regression, higher log10-transformed serum S100B concentrations remained independently associated with greater EEG abnormality severity after adjustment for gestational age and RDS (adjusted OR 19.95, 95% CI 4.97–80.16; *p* < 0.001). Higher gestational age was independently associated with lower odds of greater EEG abnormality severity (adjusted OR 0.79 per week, 95% CI 0.68–0.92; *p* = 0.003), whereas RDS was not independently associated with EEG severity (adjusted OR 2.05, 95% CI 0.80–5.25; *p* = 0.135). The proportional odds assumption was not violated (Test of Parallel Lines: χ^2^(3) = 5.810, *p* = 0.121).

### 3.3. Sensitivity Analysis Using a Preterm-Specific EEG Grading System

To assess the robustness of the association between serum S100B and EEG abnormalities using a preterm-specific grading approach, a sensitivity analysis was performed in infants with a PMA of ≤36 weeks at the time of EEG recording. EEG recordings in this subgroup (n = 89) were reassessed according to the standardized PMA-dependent grading system described by Pavlidis et al. (2020) [17].

Among the 89 infants included in the sensitivity analysis, 11 (12.4%) had grade 0 EEG abnormalities, 38 (42.7%) had grade 1, 33 (37.1%) had grade 2, 5 (5.6%) had grade 3, and 2 (2.2%) had grade 4. For subsequent analyses, the grades were grouped into three severity categories: normal/mild (grades 0–1; n = 49, 55.1%), moderate (grade 2; n = 33, 37.1%), and severe (grades 3–4; n = 7, 7.9%).

Higher serum S100B concentrations were significantly associated with increasing EEG abnormality severity according to the Pavlidis grading system (Spearman’s ρ = 0.54, *p* < 0.001). Log10-transformed S100B concentrations also differed significantly across the three EEG severity categories (Kruskal–Wallis H(2) = 25.78, *p* < 0.001). Median log10(S100B) concentrations were 1.841 (IQR 1.750–1.958) in the normal/mild group, 2.130 (IQR 1.987–2.364) in the moderate group, and 2.397 (IQR 2.016–2.602) in the severe group. Dunn’s post-hoc test with Holm correction showed significantly higher S100B concentrations in the moderate and severe groups compared with the normal/mild group (both adjusted *p* < 0.001), whereas the difference between the moderate and severe groups was not statistically significant (adjusted *p* = 0.298).

The sensitivity analysis showed a significant association between higher serum S100B concentrations and increasing EEG abnormality severity according to the Pavlidis preterm-specific grading system (Spearman’s ρ = 0.54, *p* < 0.001; Kruskal–Wallis H(2) = 25.78, *p* < 0.001; Table 5). Dunn’s post-hoc test with Holm correction showed significant differences between the normal/mild and moderate groups and between the normal/mild and severe groups (both adjusted *p* < 0.001), whereas the moderate and severe groups did not differ significantly (adjusted *p* = 0.298). These findings were consistent with the primary analysis based on the Murray classification, supporting the robustness of the association between higher serum S100B concentrations and greater EEG abnormality severity across the two EEG grading approaches.

### 3.4. Association of S100B and Structural Brain Abnormalities

Structural brain abnormalities were assessed using serial CUS, with the highest confirmed IVH grade according to the Papile classification and PVL grade according to the de Vries classification used for the analyses. Serum S100B concentrations were not significantly associated with IVH severity (Spearman’s ρ = 0.059, *p* = 0.532), and log10-transformed S100B concentrations did not differ significantly across IVH severity categories (Kruskal–Wallis H(2) = 2.72, *p* = 0.257). Similarly, no significant association was observed between S100B concentrations and PVL severity (Spearman’s ρ = −0.024, *p* = 0.797), with no significant differences across PVL severity categories (Kruskal–Wallis H(2) = 1.08, *p* = 0.583).

In univariable ordinal logistic regression analyses, several measures of prematurity and neonatal morbidity were associated with IVH severity, including gestational age, birth weight, birth length, Apgar scores, duration of IMV, RDS, and several neonatal complications (Table 6). For PVL severity, significant associations were observed with gestational age, birth weight, birth length, Apgar scores, and neonatal pneumonia. Serum S100B was not significantly associated with either IVH or PVL severity in the univariable analyses.

Given the small numbers of infants in the severe IVH and PVL categories, separate parsimonious multivariable ordinal logistic regression models were constructed for each structural outcome, including log10-transformed S100B and gestational age (Table 7).

In the multivariable analyses, serum S100B concentrations were not independently associated with either IVH severity (adjusted OR 0.60, 95% CI 0.16–2.17; *p* = 0.434) or PVL severity (adjusted OR 0.43, 95% CI 0.10–1.85; *p* = 0.256). In contrast, higher gestational age was independently associated with lower severity of both IVH (adjusted OR 0.58 per week, 95% CI 0.48–0.71; *p* < 0.001) and PVL (adjusted OR 0.75 per week, 95% CI 0.65–0.87; *p* < 0.001). Overall, these findings did not demonstrate an association between serum S100B concentrations measured in the early neonatal period and the severity of ultrasound-defined IVH or PVL.

## 4. Discussion

The principal finding of this prospective cohort study was that higher serum S100B concentrations measured within the first 24 h of life were associated with greater EEG abnormality severity at 10–14 days in preterm infants. This association remained significant after adjustment for gestational age and RDS and was reproduced in a PMA-restricted sensitivity analysis using a preterm-specific EEG grading approach. Although the association remained statistically significant in the multivariable analysis (OR = 19.95, 95% CI: 4.97–80.16), the wide confidence interval indicates uncertainty regarding its magnitude and warrants cautious interpretation of the effect size. In contrast, S100B was not significantly associated with the severity of IVH or PVL assessed by serial CUS, including after adjustment for gestational age. Thus, our findings are consistent with the working hypothesis that early circulating S100B is associated with neurophysiological abnormalities in preterm infants, whereas no significant association with ultrasound-defined IVH or PVL was observed during the early neonatal period evaluated in this study.

The association between S100B and EEG severity should be considered in the context of previous studies demonstrating the clinical relevance of neonatal EEG background abnormalities [18,19,20]. Murray et al. (2009) prospectively studied term infants with hypoxic–ischemic encephalopathy using continuous multichannel video-EEG initiated within 6 h after birth and continued for up to 72 h; 44 infants completed neurodevelopmental follow-up at 24 months [16]. EEG background severity was associated with later neurodevelopmental outcome. Although their population differed substantially from our preterm cohort, the study demonstrated the clinical relevance of graded neonatal EEG background abnormalities as indicators of cerebral dysfunction. Our findings complement these observations by demonstrating an association between EEG abnormality severity and an early circulating glial biomarker in preterm infants. However, because neurodevelopmental outcomes were not assessed in our study, the S100B–EEG association cannot be assigned the prognostic significance demonstrated for EEG abnormalities in the Murray cohort.

An important methodological consideration was the broad range of cerebral maturation at the time of EEG assessment. Although all infants were born preterm, some had reached a PMA > 36 weeks by the time EEG was performed at 10–14 days of life. Consequently, no single EEG grading system was ideally applicable to the entire cohort. The Murray approach was originally developed in term infants with hypoxic–ischemic encephalopathy, whereas Pavlidis et al. (2020) developed a standardized scheme incorporating maturational characteristics of preterm EEG [17]. Their methodological study initially evaluated recordings from 24 infants born at <32 weeks of gestation and subsequently tested the scheme in an additional 12 infants born at <37 weeks, covering PMA from 23–25 to 35–36 weeks. We therefore used the Murray classification for the primary analysis and the PMA-specific Pavlidis approach for sensitivity analysis in the 89 infants with PMA ≤ 36 weeks at EEG assessment. The association between S100B and EEG severity persisted in this subgroup (Spearman’s ρ = 0.54, *p* < 0.001), supporting the robustness of the principal finding across different EEG grading approaches. The two systems should not be considered interchangeable; rather, the sensitivity analysis provides complementary evidence while accounting more explicitly for maturational EEG characteristics.

The importance of maturationally appropriate EEG interpretation in preterm infants is also supported by Lloyd et al. (2021), who prospectively studied infants born at <32 weeks of gestation [18]. Seventy infants were enrolled, 67 were included in the final cohort, and neurodevelopmental outcome at 2 years’ corrected age was available for 57. Serial age-specific EEG abnormalities were associated with later neurodevelopmental outcome, with particularly strong discrimination at approximately 35 weeks PMA. These findings support the clinical relevance of maturationally appropriate EEG assessment in preterm infants [18,19]. Our study addresses a different question but adds evidence that early circulating S100B is associated with subsequent EEG abnormality severity. This association is biologically plausible because S100B is predominantly expressed by astroglial cells and has been investigated as a circulating marker of cerebral injury in neonates [7]. However, circulating S100B is not specific to cerebral injury and may be influenced by gestational maturity, systemic illness, extracerebral sources, and sampling time. In our study, the S100B–EEG association remained significant after adjustment for gestational age and RDS, suggesting that it was not explained solely by differences in maturity or early respiratory morbidity. Nevertheless, the observational design does not establish causality or identify the biological mechanism underlying this association.

In contrast to the EEG findings, S100B was not significantly associated with IVH or PVL severity. Similar findings were reported by Koce et al. (2022) in a prospective pilot study of 40 very preterm infants born at 23–32 weeks of gestation [21]. Serum S100B was measured within the first 24 h of life and again at 4–7 days, while brain injury was assessed by CUS. Seven infants had IVH and/or PVL, but S100B concentrations did not differ significantly between infants with and without these abnormalities. Although their cohort was smaller than ours and the detected structural abnormalities were predominantly mild, these findings are consistent with the absence of a significant association between early S100B and IVH or PVL in our study. Differences in lesion type and severity, timing of biomarker sampling and neuroimaging, and the sensitivity of the imaging modality may influence the relationship between circulating S100B and structural brain abnormalities.

Importantly, the different findings for EEG and CUS should not be interpreted as evidence that S100B detects functional disturbances before structural abnormalities become apparent. EEG and CUS assess different aspects of cerebral status: EEG provides a dynamic assessment of cerebral function and background organization, whereas CUS primarily identifies structural abnormalities such as IVH and evolving white matter injury [11,18]. In our study, CUS was initiated on the first day of life and repeated every 3 days through 10–14 days, with the highest confirmed IVH and PVL grades used for analysis. Grade I PVL required abnormal periventricular echogenicity persisting for at least 7 days. Nevertheless, some forms of structural brain injury may become apparent later or may be more readily detected by MRI [11,22]. Therefore, the absence of an association with IVH or PVL does not exclude a relationship between early S100B and structural abnormalities developing later or detectable with more sensitive neuroimaging methods [11,22].

The temporal sequence of the findings also requires cautious interpretation. S100B measurement within the first 24 h preceded EEG assessment at 10–14 days, whereas CUS was performed serially throughout the same early neonatal period. The present study therefore demonstrates an association between early S100B and subsequent EEG severity, but it does not establish a temporal progression from biochemical alteration to functional disturbance and later structural brain injury. The timing and design of the assessments do not allow us to establish a consistent temporal sequence between EEG abnormalities and structural injury, nor to determine whether early S100B predicts structural abnormalities developing later. Longer neuroimaging follow-up, including assessment at term-equivalent age, would be required to address these relationships. Accordingly, the present findings should not be interpreted as evidence that S100B predicts later structural brain injury.

The clinical significance of the observed S100B–EEG association remains to be established in longitudinal studies. Previous studies have demonstrated associations between neonatal EEG abnormalities and later neurodevelopmental outcomes, including the study by Murray et al. in term infants with hypoxic–ischemic encephalopathy and the prospective study by Lloyd et al. in very preterm infants [16,18]. However, these findings cannot be directly extrapolated to circulating S100B. In the absence of neurodevelopmental follow-up in the present cohort, it remains unknown whether higher early S100B concentrations or the associated EEG abnormalities are related to subsequent neurological or neurodevelopmental impairment. Accordingly, S100B should not be interpreted as a prognostic biomarker on the basis of the present findings.

Overall, our findings support further investigation of S100B as a potential component of multimodal neurological assessment rather than as a standalone diagnostic or prognostic biomarker. Biochemical, electrophysiological, and neuroimaging measures may provide complementary information on different aspects of cerebral status in preterm infants. Future prospective longitudinal studies combining serial S100B measurements, PMA-specific EEG assessment, longer CUS follow-up, term-equivalent MRI, and standardized neurodevelopmental assessment are needed to determine the temporal and prognostic significance of the observed S100B–EEG association and its potential clinical relevance.

## 5. Limitations

This study has several limitations. First, its observational design precludes causal inference, and residual confounding related to prematurity and neonatal morbidity cannot be excluded. Second, S100B was measured at a single early time point, precluding assessment of temporal changes in biomarker concentrations and their relationship with evolving neurological abnormalities. Third, CUS follow-up was limited to the first 10–14 days of life, and severe structural abnormalities were uncommon, with only six infants having severe IVH and five having severe PVL. This limited the precision of the structural analyses and does not exclude abnormalities developing later in the neonatal period. Fourth, neurological assessment was based on EEG and CUS without MRI, which may have limited the detection of subtle or diffuse structural brain injury. Finally, long-term neurodevelopmental outcomes were not assessed. Consequently, the present study cannot determine whether early S100B concentrations or the associated EEG abnormalities are related to later neurodevelopmental impairment.

## 6. Conclusions

Higher serum S100B concentrations measured within the first 24 h of life were associated with greater subsequent EEG abnormality severity in preterm infants, and this association remained significant after adjustment for gestational age and RDS. The association was also reproduced in a PMA-specific sensitivity analysis using a preterm EEG grading approach. In contrast, no significant association was observed between early S100B concentrations and the severity of IVH or PVL detected by CUS during the early neonatal period. These findings demonstrate an association between early S100B and subsequent neurophysiological abnormalities but do not establish a temporal progression to structural brain injury or predict later neurodevelopmental impairment. Longitudinal studies incorporating serial biomarker measurements, later neuroimaging, and neurodevelopmental follow-up are required to determine the temporal and prognostic significance of the observed S100B–EEG association.

## Figures and Tables

**Table 1:** Baseline characteristics of infants with and without EEG assessment.

Variable	EEG Group (n = 116)	No EEG Group (n = 165)	Mann–Whitney U Test	*p*
Gestational age, weeks	34 (32–35)	33 (32–35)	10,341.0	0.245
Birth weight, g	2260 (1677.5–2502.5)	2100 (1690–2490)	10,050.0	0.475
Birth length, cm	45 (42–46)	44 (42–46)	10,317.5	0.259
Apgar score at 1 min	6 (5–6)	6 (5–6)	9386.5	0.774
Apgar score at 5 min	7 (6–7)	7 (7–7)	9075.0	0.408

**Table 2:** Clinical characteristics and neurological outcomes of the final analytical cohort (n = 116).

Variable	Value
Perinatal and respiratory characteristics	
Sex, male/female	61/55 (52.6%/47.4%)
Surfactant administration	45 (38.8%)
Maximum respiratory support:	
none	20 (17.2%)
CPAP	59 (50.9%)
IMV	37 (31.9%)
Duration of IMV, days, median (IQR)	0 (0–2.25)
Respiratory distress syndrome (RDS)	80 (69.0%)
Bronchopulmonary dysplasia (BPD)	12 (10.3%)
Pneumonia (congenital and/or postnatal)	68 (58.6%)
Neonatal sepsis (early- and/or late-onset)	4 (3.4%)
Necrotizing enterocolitis (NEC)	2 (1.7%)
Retinopathy of prematurity (ROP)	3 (2.6%)
Anemia of prematurity (AOP)	21 (18.1%)
Disseminated intravascular coagulation (DIC)	11 (9.5%)
Neurological outcomes	
IVH severity (Papile classification):	
no IVH	46 (39.7%)
mild IVH (grades I–II)	64 (55.2%)
severe IVH (grades III–IV)	6 (5.2%)
PVL grade (de Vries classification):	
no PVL	90 (77.6%)
mild PVL (grades I–II)	21 (18.1%)
severe PVL (grades III–IV)	5 (4.3%)
EEG background abnormality severity (Murray et al., 2009 [16]):	
normal/mild (grades 0–1)	15 (12.9%)
moderate (grade 2)	58 (50.0%)
severe/inactive–ictal (grades 3–4)	43 (37.1%)
EEG severity (Pavlidis et al., 2020 [17]; PMA ≤ 36 weeks, n = 89):	
normal/mild (grades 0–1)	49 (55.1%)
moderate (grade 2)	33 (37.1%)
severe (grades 3–4)	7 (7.9%)

**Table 3:** Univariable ordinal logistic regression analyses of factors associated with EEG abnormality severity.

Variable	OR	95% CI	*p* Value
S100B, log10	26.04	6.75–100.49	<0.001
Gestational age, per week	0.71	0.60–0.83	<0.001
Birth weight, per 100 g	0.92	0.87–0.97	0.001
Birth length, cm	0.90	0.83–0.96	0.003
Male sex	0.71	0.35–1.42	0.332
Apgar score at 1 min	0.68	0.53–0.89	0.005
Apgar score at 5 min	0.66	0.48–0.92	0.014
Cesarean delivery	0.45	0.17–1.24	0.123
Duration of IMV, per day	1.12	1.02–1.23	0.017
RDS	4.22	1.86–9.60	<0.001
BPD	2.95	0.91–9.56	0.072
Neonatal pneumonia	1.85	0.90–3.82	0.096
Neonatal sepsis	NE ^1^	NE	NE
NEC	NE	NE	NE
AOP	1.74	0.72–4.21	0.219
DIC	2.51	0.76–8.33	0.133
ROP	3.74	0.34–40.69	0.279

^1^ NE, not estimable because of sparse events.

**Table 4:** Multivariable ordinal logistic regression analysis of factors associated with EEG background abnormality severity.

Variable	β	SE	Adjusted OR	95% CI	*p*
S100B, log10	2.993	0.709	19.95	4.97–80.16	<0.001
Gestational age, per week	−0.237	0.079	0.79	0.68–0.92	0.003
RDS, yes vs. no	0.718	0.480	2.05	0.80–5.25	0.135

The proportional odds assumption was assessed using the Test of Parallel Lines and was not violated (χ^2^(3) = 5.810, *p* = 0.121).

**Table 5:** Association between serum S100B concentrations and EEG abnormality severity according to the Pavlidis preterm-specific grading system.

EEG Severity	n (%)	Median log10(S100B)	IQR
Normal/mild (grades 0–1)	49 (55.1)	1.841 ^a^	1.750–1.958
Moderate (grade 2)	33 (37.1)	2.130 ^b^	1.987–2.364
Severe (grades 3–4)	7 (7.9)	2.397 ^b^	2.016–2.602

Values with different superscript letters indicate statistically significant pairwise differences based on Dunn’s post-hoc test with Holm correction.

**Table 6:** Univariable ordinal logistic regression analyses of clinical factors associated with IVH and PVL severity.

Variable	IVH Severity (Papile)			PVL Severity (de Vries)		
	OR	95% CI	*p* Value	OR	95% CI	*p* Value
S100B, log10	1.73	0.58–5.16	0.328	0.73	0.19–2.76	0.645
Gestational age, per week	0.59	0.49–0.71	<0.001	0.76	0.66–0.88	<0.001
Birth weight, per 100 g	0.77	0.70–0.84	<0.001	0.89	0.83–0.96	0.002
Birth length, per cm	0.71	0.64–0.80	<0.001	0.89	0.82–0.96	0.004
Male sex	0.85	0.41–1.75	0.658	0.82	0.34–1.96	0.654
Apgar score at 1 min	0.33	0.23–0.50	<0.001	0.56	0.42–0.75	<0.001
Apgar score at 5 min	0.23	0.13–0.39	<0.001	0.52	0.37–0.74	<0.001
Cesarean delivery	3.08	1.05–9.07	0.041	0.99	0.30–3.31	0.992
Duration of IMV, per day	1.16	1.06–1.28	0.002	1.05	0.98–1.13	0.177
RDS	9.88	3.98–24.54	<0.001	1.76	0.64–4.81	0.273
BPD	4.26	1.18–15.45	0.027	2.40	0.73–7.91	0.151
Neonatal pneumonia	5.28	2.39–11.70	<0.001	3.05	1.12–8.28	0.029
Neonatal sepsis	29.00	3.40–247.28	0.002	2.93	0.46–18.78	0.258
NEC	23.33	1.37–396.14	0.029	2.84	0.21–37.80	0.428
AOP	3.95	1.39–11.16	0.010	2.09	0.74–5.85	0.162
DIC	17.63	3.61–86.02	<0.001	1.23	0.31–4.93	0.765
ROP	12.93	1.24–134.56	0.032	1.59	0.15–17.09	0.703

**Table 7:** Multivariable ordinal logistic regression analyses of factors associated with IVH and PVL severity.

Variable	β	SE	Adjusted OR	95% CI	*p*
IVH severity (Papile classification)					
S100B, log10	−0.517	0.661	0.60	0.16–2.17	0.434
Gestational age, per week	−0.542	0.100	0.58	0.48–0.71	<0.001
PVL severity (de Vries classification)					
S100B, log10	−0.848	0.746	0.43	0.10–1.85	0.256
Gestational age, per week	−0.291	0.074	0.75	0.65–0.87	<0.001

Separate ordinal logistic regression models were fitted for IVH and PVL severity. The proportional odds assumption was not significantly violated for either model (IVH: Test of Parallel Lines, χ^2^(2) = 5.338, *p* = 0.069; PVL: χ^2^(2) = 2.146, *p* = 0.342).

## Data Availability

The data supporting the findings of this study can be provided by the corresponding author upon reasonable request.

## Abbreviations

The following abbreviations are used in this manuscript:

Abbreviation	Definition
AOP	Anemia of prematurity
BPD	Bronchopulmonary dysplasia
CI	Confidence interval
CPAP	Continuous positive airway pressure
CUS	Cranial ultrasonography
DIC	Disseminated intravascular coagulation
EEG	Electroencephalography
IMV	Invasive mechanical ventilation
IQR	Interquartile range
IVH	Intraventricular hemorrhage
MRI	Magnetic resonance imaging
NE	Not estimable
NEC	Necrotizing enterocolitis
NICU	Neonatal intensive care unit
OR	Odds ratio
PMA	Postmenstrual age
PVL	Periventricular leukomalacia
RDS	Respiratory distress syndrome
ROP	Retinopathy of prematurity
SE	Standard error
